# Analysis of Animal Research Ethics Committee Membership at American Institutions

**DOI:** 10.3390/ani2010068

**Published:** 2012-02-22

**Authors:** Lawrence A. Hansen, Justin R. Goodman, Alka Chandna

**Affiliations:** 1 Departments of Neurosciences and Pathology, University of California San Diego, 9500 Gilman Drive, La Jolla, CA 92093, USA; E-Mail: lahansen@ucsd.edu; 2 People for the Ethical Treatment of Animals, 1536 16th Street NW, Washington, DC 20036, USA; E-Mail: alkac@peta.org; 3 Department of Sociology and Criminal Justice, Marymount University, 2807 N. Glebe Road, Arlington, VA 22207, USA

**Keywords:** animal research, ethics, laboratory animals, Institutional Animal Care and Use Committees, 3Rs

## Abstract

**Simple Summary:**

This study analyzed the membership of animal experimentation oversight committees at leading U.S. research institutions. We found the leadership and general membership of these committees to be dominated by animal researchers and the remainder of the committees to be largely comprised of other institutional representatives. These arrangements may contribute to previously-documented committee biases in favor of approving animal experiments and dilute input from the few members representing animal welfare and the interests of the general public.

**Abstract:**

Institutional Animal Care and Use Committees (IACUCs) were created to review, approve and oversee animal experiments and to balance the interests of researchers, animals, institutions and the general public. This study analyzed the overall membership of IACUCs at leading U.S. research institutions. We found that these committees and their leadership are comprised of a preponderance of animal researchers, as well as other members who are affiliated with each institution; some of whom also work in animal laboratories. This overwhelming presence of animal research and institutional interests may dilute input from the few IACUC members representing animal welfare and the general public, contribute to previously-documented committee bias in favor of approving animal experiments and reduce the overall objectivity and effectiveness of the oversight system.

## 1. Introduction

In response to growing public concerns about the welfare of animals in laboratories stemming from exposés of abuse at several high-profile federally-funded research facilities, in 1985 U.S. Congress and the Public Health Service (PHS) mandated the institutional animal care and use committee (IACUC) system to oversee vertebrate animal use and ensure compliance with federal regulations and guidelines [1]. Lawmakers envisioned IACUCs—which are charged with reviewing, approving and regularly monitoring animal use—to be the cornerstone of humane treatment of animals in laboratories at each federally-regulated and -funded facility.

Because of the important role these committees play, there has been significant research interest in the way IACUCs function in practice. Many studies have explored the subjective experiences of IACUC members and documented the dynamic process of how full committees deliberate protocols under review [2,3,4,5,6,7,8,9]. Among other insights, this work has found great variability in individual IACUC members’ interpretation of what constitutes the ethics of animal research, what their personal roles on the committee are and what the committee’s overall responsibilities and authority are. Researchers have also described how these committees’ decision-making is significantly shaped by personal and political dynamics among members.

Some analyses have focused more narrowly on the outcomes of protocol reviews by IACUCs [3,10,11] and similarly reflect the subjectivity of the oversight system. One seminal study by Plous and Herzog [10] found large inconsistencies between reviews of the same protocols by IACUCs at 50 different U.S. institutions, with IACUCs rating the quality of their own protocols much more favorably than reviewers from other facilities. The authors concluded that, “IACUCs will rarely disapprove of protocols that other committees feel should be rejected.” Committees examined for that study approved 98 percent of their own protocols, and other papers have reported similarly high IACUC approval rates [3,5,8,9,10]. Federal audits [12], government surveys of U.S. Department of Agriculture (USDA) laboratory inspectors [13], and USDA reports [14,15] have also found that IACUCs frequently approve protocols that fail to meet federal standards.

Taken together these data appear to illustrate that, despite any individual differences among their committee members, IACUCs ultimately may have a bias in favor of approving the protocols they review. This speaks to the power of the IACUC as a whole and identifies overall committee composition as a subject warranting further analysis.

Researchers looking at IACUC functioning at several universities in Canada—whose committees operate and are constituted similarly to those in the U.S.—have attributed some of the acceptance bias in the protocol review process to the fact that IACUCs are often disproportionately comprised of people who are affiliated with the institution and who use animals [8,9]. In the U.S., while minimum IACUC membership requirements are stipulated by PHS Policy and the Animal Welfare Act (AWA) (Table 1) and at their foundation equally represent all interests (animal researchers, the institution, animals and the public), IACUCs in the U.S.—unlike other countries—are not required to maintain a balance between these various members. As a result, we have previously observed through our own interactions with U.S. IACUCs regarding animal welfare issues and regular reviews of IACUC-produced documents that animal researchers often greatly outnumber other members.

The purpose of this study is to systematically evaluate the overall membership composition of IACUCs at leading U.S. research institutions, which has not been done in previous research.

## 2. Materials and Methods

### 2.1. Sample

The current study examined IACUC membership at the top 25 National Institutes of Health (NIH)-funded research institutions, which was based on the total value of all NIH grants awarded in 2010 [16].

### 2.2. Data Collection

Using the federal Freedom of Information Act and state open records requests, IACUC rosters were obtained from Animal Welfare Assurances that each facility filed with the NIH’s Office of Laboratory Animal Welfare (OLAW) as of September 2010. Four facilities were excluded from the final analysis because their Assurances did not include information about the roles of more than 25 percent of their IACUC members.

**Table 1:** Minimum IACUC membership requirements set forth by the Public Health Service and USDA.

PHS Policy [17]	Animal Welfare Act [18]
Five members, including: ■ One Doctor of Veterinary Medicine with training or experience in laboratory animal science and medicine who has direct or delegated program authority and responsibility for activities involving animals at the institution. ■ One practicing scientist experienced in research involving animals. ■ One member whose primary concerns are in a nonscientific area (for example, ethicist, lawyer, clergy). ■ One member not affiliated in any way with the institution and not a member of the immediate family of a person who is affiliated with the institution.	Three members, including: ■ Chairperson ■ At least one Doctor of Veterinary Medicine with training or experience in laboratory animal science and medicine, and who has direct or delegated program responsibility for activities involving animals at the institution. ■ One member not affiliated in any way with the institution and not a member of the immediate family of a person who is affiliated with the institution; person who represents the general community interests in the proper care and treatment of animals; and is not a laboratory animal user

### 2.3. Data Organization

IACUC members were classified into the four categories defined by PHS: scientists, non-scientists, unaffiliated members, and veterinarians (Table 1). Those designated as “scientists” are, by PHS definition, people who use animals in research, while “non-scientists” and “unaffiliated” members do not (the latter groups can be trained scientists, despite the PHS nomenclature). Although many veterinarians serving on IACUCs are involved in animal research, PHS categorizes them separately from animal researchers because in principal their primary role on the IACUC is to provide oversight and expertise on matters of animal health and welfare.

Several IACUCs placed individuals in two membership categories, as is permitted by PHS Policy under certain conditions [17]. Where an IACUC counted one member as both nonaffiliated and nonscientist, we counted them as a nonaffiliated member because under PHS definition, these members must not be animal researchers. This allowed us to ensure that each member was only counted once in our analysis and to distinguish between nonscientists who were affiliated with the institutions and those who were not.

## 3. Results and Discussion

Our review of IACUC membership at the 21 institutions examined in our study revealed that their composition was indeed imbalanced (Table 2). The mean size of these IACUCs was 23 members (SD = 14.24). Our analysis revealed that on average 66.81 percent (SD = 12.05) of the committees were comprised of animal researchers, 15.05 percent (SD = 6.41) were veterinarians, 9.86 percent (SD = 6.58) were nonaffiliated individuals, and 8.28 percent (SD = 5.52) were non-scientists. Our analysis also revealed that 92.6 percent of IACUC chairpersons were animal researchers.

Because PHS Policy does not require that the distinction be made and certain identifying information about IACUC members was redacted from the documents examined for this study, it was not possible to accurately determine how many veterinarians on these IACUCs in addition to the Attending Veterinarian were involved in animal research. However, several IACUC rosters did contain enough information for us to ascertain that all of their veterinarian members were involved in animal research; these included the University of Pennsylvania, Washington University in St. Louis, University of California-San Francisco, University of Wisconsin-Madison and Stanford University.

**Table 2:** IACUC composition at top 25 NIH-funded institutions.

	Scientists	Veterinarians	Non-scientists	Nonaffiliated members	Undesignated members	Is chair a scientist?
**1. Baylor College of Medicine**	23	2	1(1) **	1	0	Y
**2. Columbia University Health Sciences**	15	3	3	2	0	Y
**3. Duke University**	21	4	3	1	0	N
**4. Emory University**	16	7	2	1	0	Y
**5. Fred Hutchinson Cancer Research Center**	3	1	1(1) **	2	2^#^	Y
**6. Johns Hopkins University**	15	3	1(1) **	2	0	Y
**7. Mass General Hospital**	26	7	1(1) **	2	0	Y
**8. Scripps Research Institute**	6	2	1(1)**	1	0	Y
**9. Stanford University**	5	3	1	2	0	Y
**10. University of Alabama at Birmingham **	11	4	3	0^ β^	0	Y
**11. University of California, Los Angeles**	12	2	1(2) **	2	1^#^	Y
**12. University of California, San Diego**	14	1	2	2	0	Y
**13. University of California, San Fran**	6	2	0(1) **	2	1^#^	Y
**14. University of Michigan at Ann Arbor**	10	2	2(2) **	2	0	Y
**15. University of Minnesota, Twin Cities**	13	1	0(1) **	1	3^#^	Y
**16. University of North Carolina, Chapel Hill**	7	1	2	1	0	Y
**17. University of Pennsylvania**	10	5	3	3	0	Y
**18. University of Pittsburgh at Pittsburgh**	30	4	1(1) **	2	0	Y
**19. University of Wisconsin, Madison ^†^**	48	11	8	7	0	5Y/1N^†^
**20. Vanderbilt University**	24	3	2	1	0	Y
**21. Washington University**	12	2	0(1) **	2	1^#^	Y
**x- Brigham and Women’s Hospital/Harvard Medical School**	10	2	0(2) **	2	5	Y
**x- University of Washington**	1	4	1(3) **	3	7	N
**x- Yale University**	9	2	1	1	5	Y

**^β^** Institution’s IACUC roster did not place a member in the required nonaffiliated role. ** Number in parentheses reflects individual IACUC members who were designated as both nonscientists and nonaffiliated members but were counted only as nonaffiliated members for this study. See methods for further explanation. ^#^ Data points excluded due to missing information. ^x^ Facilities excluded from final analysis due to large amount of missing data. ^†^ UW-Madison has six IACUCs that were combined for this analysis. Five of six chairs were animal researchers.

The preponderance of animal researchers on IACUCs and in their leadership positions raises serious concerns about bias in the animal research protocol review and approval process.

Because U.S. IACUCs employ a majority voting system, arithmetic alone places the ultimate authority of the committees in the hands of animal researchers. This imbalance is further magnified when considering that some of the IACUCs’ veterinarian and nonscientist members also use animals in laboratories and represent the institution’s interests, respectively. This arrangement necessarily marginalizes the input of members who speak on behalf of the public that funds much of the animal research being conducted and who are increasingly critical of the practice [19].

Relationships among animal researchers, laboratory veterinarians, and other institutional faculty and staff who comprise the overwhelming majority of IACUCs can foster an environment where IACUC members may not want to criticize the work of their peers and friends, particularly in a setting where most deliberations occur in person and voting is not anonymous. This is especially true of animal researchers and veterinarian-researchers serving on IACUCs. Both have inherent conflicts of interest because they have a personal stake in maintaining the favor of their committee colleagues so that their own protocols—the success of which are linked to publishing and funding opportunities and promotion in rank and tenure—are favorably reviewed.

Schuppli and Fraser [9] have discussed how imbalanced IACUC composition favoring researchers and institutional interests can lead to groupthink, polarization of opinions and the suppression of dissent, which could explain the exorbitant protocol approval rates that have been documented. Nonaffiliated IACUC members have reported feeling intimidated and as though their input is dismissed or diluted because they are outnumbered [2,7,9]. Other studies have found IACUC members believe that membership comprised of more nonscientists and community members would improve the committee’s functioning [4]. In Sweden [10] and Australia [20], one half and one third of animal research ethics committees, respectively, must be comprised of nonaffiliated laypersons and animal welfare experts.

Increasing the proportion of scientists who do not conduct animal research, animal welfare experts and nonaffiliated laypeople would help elevate the role of these IACUC members from one of acting as “public witness” [2] of committee deliberations to one that can more meaningfully influence laboratory policy and practice. At the least, it would ensure that IACUC deliberations and decisions better reflected the values and perspectives of the general public whose tax dollars support the work it reviews and whose interests this work supposedly serves. At best, this shift might result in more time and attention being given to seeking and considering potential alternatives which might reduce and replace animal use. It could also increase IACUCs’ discussion of fundamental ethical concerns, such as whether some animal studies—even if they conform to federal regulations—should be approved at all.

## 4. Conclusions

The NIH has acknowledged that “the validity of IACUC actions is always predicated on the existence of a properly constituted IACUC” [21]. While this comment referred to facilities’ ability to conform to the minimum IACUC membership requirements clearly this holds true for IACUC membership more broadly as well. IACUCs that choose to appoint overwhelming proportions of animal researchers, laboratory animal veterinarians and other members with vested interests in seeing animal experiments approved make committee bias likely. This imbalance also may undermine public confidence in the objectivity of the animal research review process and contribute to deficiencies in IACUC oversight that have been documented and that may result in unnecessary animal use and suffering [22].

The present study looked at only a small sample of large, high-profile institutions conducting animal research. Future researchers could seek to determine if these results reflect the makeup of U.S. IACUCs more broadly and whether committee composition and IACUC protocol review outcomes are correlated.
